# Endoscopic Removal of Large Sharp-edged Foreign Bodies in the Gastrointestinal Tract Using an Innovative Modification of the Overtube

**DOI:** 10.7759/cureus.3264

**Published:** 2018-09-06

**Authors:** Faisal Inayat, Fahad Zafar, Hanan T Lodhi, Maham Hayat, Hafiz M. Kashif Saleem, Arslan Afzal, Chaudhry Saad Sohail

**Affiliations:** 1 Internal Medicine, Allama Iqbal Medical College, Lahore, PAK; 2 Internal Medicine, King Edward Medical University, Lahore, PAK; 3 Infectious Disease, University of Nebraska Medical Center, Omaha, USA; 4 Medicine, University of Oklahoma Health Sciences Center, Oklahoma, USA; 5 Department of Medicine, Sharif Medical and Dental College, Lahore, PAK; 6 Internal Medicine, Banner University Medical Center, Tucson, USA

**Keywords:** gastrointestinal tract, sharp-edged foreign body, therapeutic endoscopy, overtube

## Abstract

Foreign body ingestion is commonly encountered in clinical practice. According to standard guidelines, urgent therapeutic endoscopy should be performed in cases involving sharp objects to prevent complications. Although several extraction methods are available, few cases may still pose a therapeutic challenge. This report describes a novel endoscopic technique utilizing modification of the standard overtube to facilitate the removal of a large razor blade. This technique offers a minimally invasive approach for rapid retrieval of large sharp-edged foreign bodies, obviating the need for a surgical exploration. Additionally, this article compares various imaging modalities for prompt detection of gastrointestinal foreign bodies to avoid unnecessary delays in endoscopic intervention.

## Introduction

Foreign body impaction or ingestion is a frequently encountered clinical entity. Although most foreign bodies eventually pass through the digestive tract, gastroenterologists should recognize situations where urgent endoscopic interventions are warranted [1]. In such cases, removal of an ingested sharp-edged object can pose a therapeutic challenge. The flexible endoscope, a protector hood or overtube are various options for protection of the gastrointestinal tract during retrieval of foreign bodies [2]. An overtube is a sleeve-like device with a diameter larger than that of an endoscope, designed to facilitate endoscopic removal of sharp objects by mucosal protection of the digestive tract [3]. The present case signifies the importance of an improvised modification of the overtube. While a standard overtube precludes removal of the foreign bodies larger than 16.7 mm in size, this unusual alteration may facilitate withdrawal of relatively larger sharp-edged objects, without any procedural complications. Furthermore, this article reviews the pertinent medical literature regarding the diagnosis and management of patients with gastrointestinal foreign bodies.

## Case presentation

A 43-year-old male presented to the emergency department with worsening abdominal pain due to ingestion of a razor blade one week ago. His past medical history was significant for schizophrenia, treated with haloperidol decanoate 250 mg per month. However, he demonstrated poor treatment adherence and received his last dose two months ago. The patient had no history of dysphagia, food impaction, or gastrointestinal surgery. He did not report any difficulty breathing. Upon evaluation, he was hemodynamically stable. His blood pressure was 126/84 mm Hg, heart rate 85 beats per minute, temperature 37.9°C, respiratory rate 16 per minute, and oxygen saturation 98% on room air. Physical and abdominal examinations were unremarkable. No blood was noticed in the rectal vault on the digital rectal examination.

**Figure 1:**
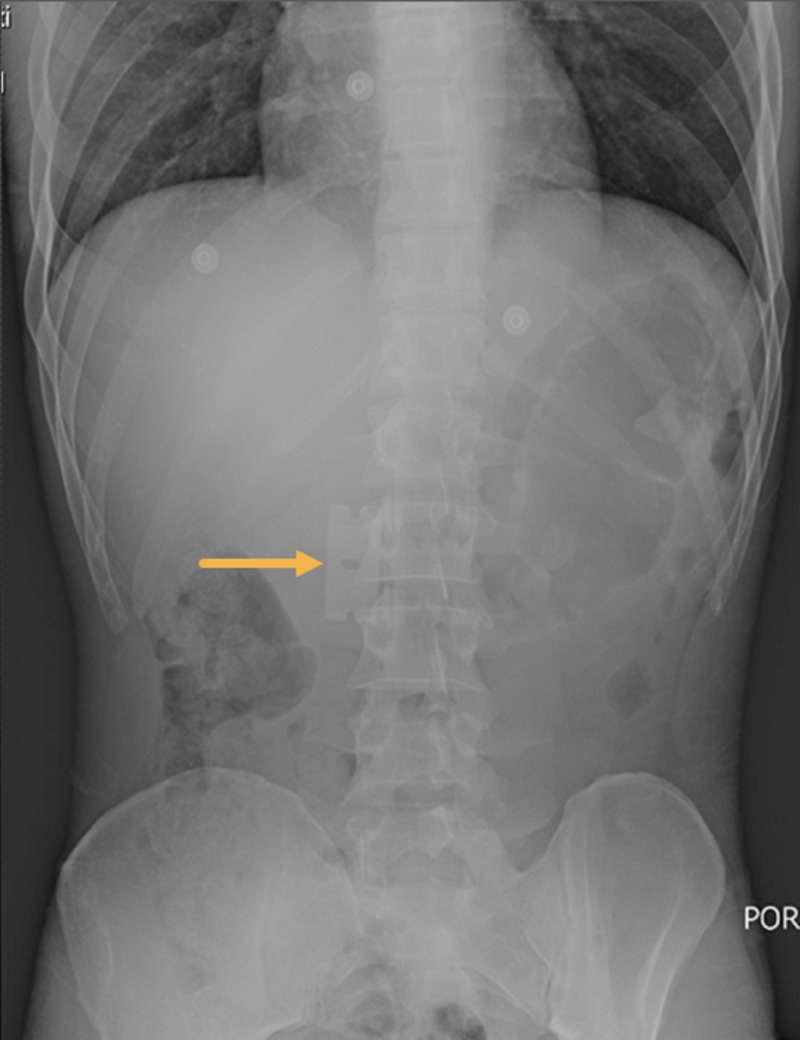
Plain abdominal radiograph showing a razor blade overlying L2 vertebral body likely in the duodenum, with no pneumoperitoneum. Arrow indicates the foreign body.

A plain abdominal radiograph showed a razor blade overlying the L2 vertebral body in the duodenal location, measuring approximately 45 x 22 mm with no evidence of bowel obstruction or pneumoperitoneum (Figure 1).

**Figure 2:**
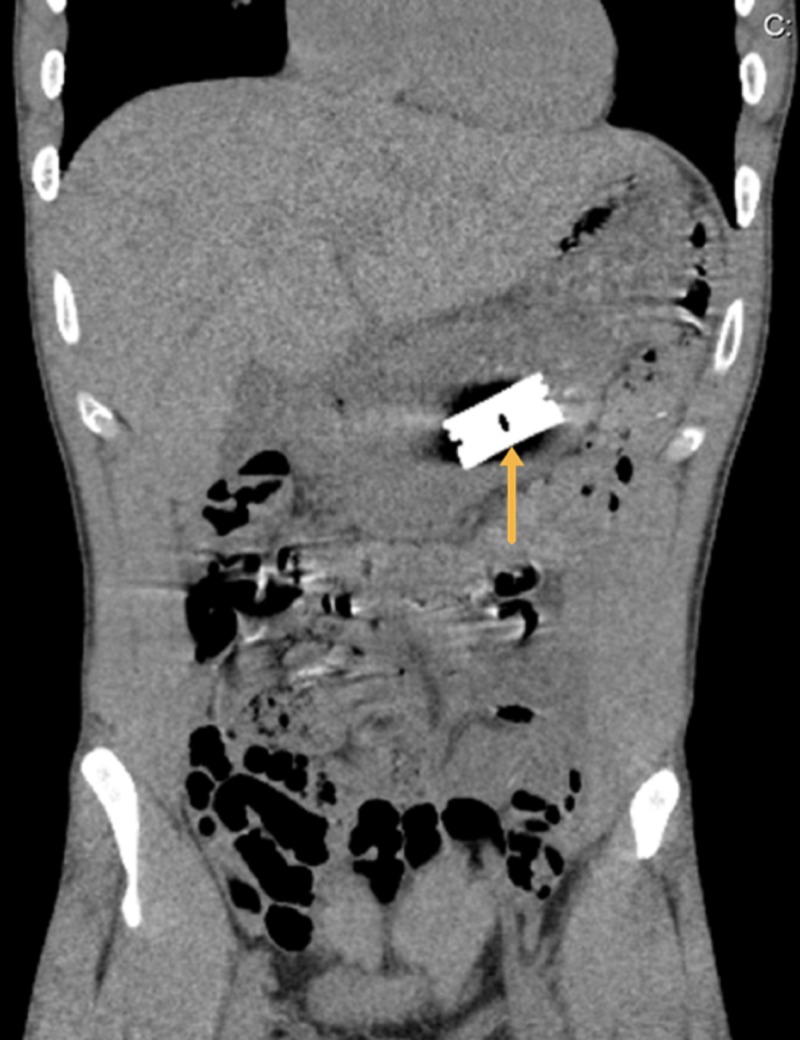
Computed tomography scan of the abdomen identifying the razor blade within the lumen of the stomach. Arrow demarcates the precise location of the razor blade.

However, the precise location of the foreign body in the gastrointestinal tract could not be determined. Therein, a computed tomography (CT) scan of the abdomen identified the razor blade within the lumen of the stomach (Figure 2).

**Figure 3:**
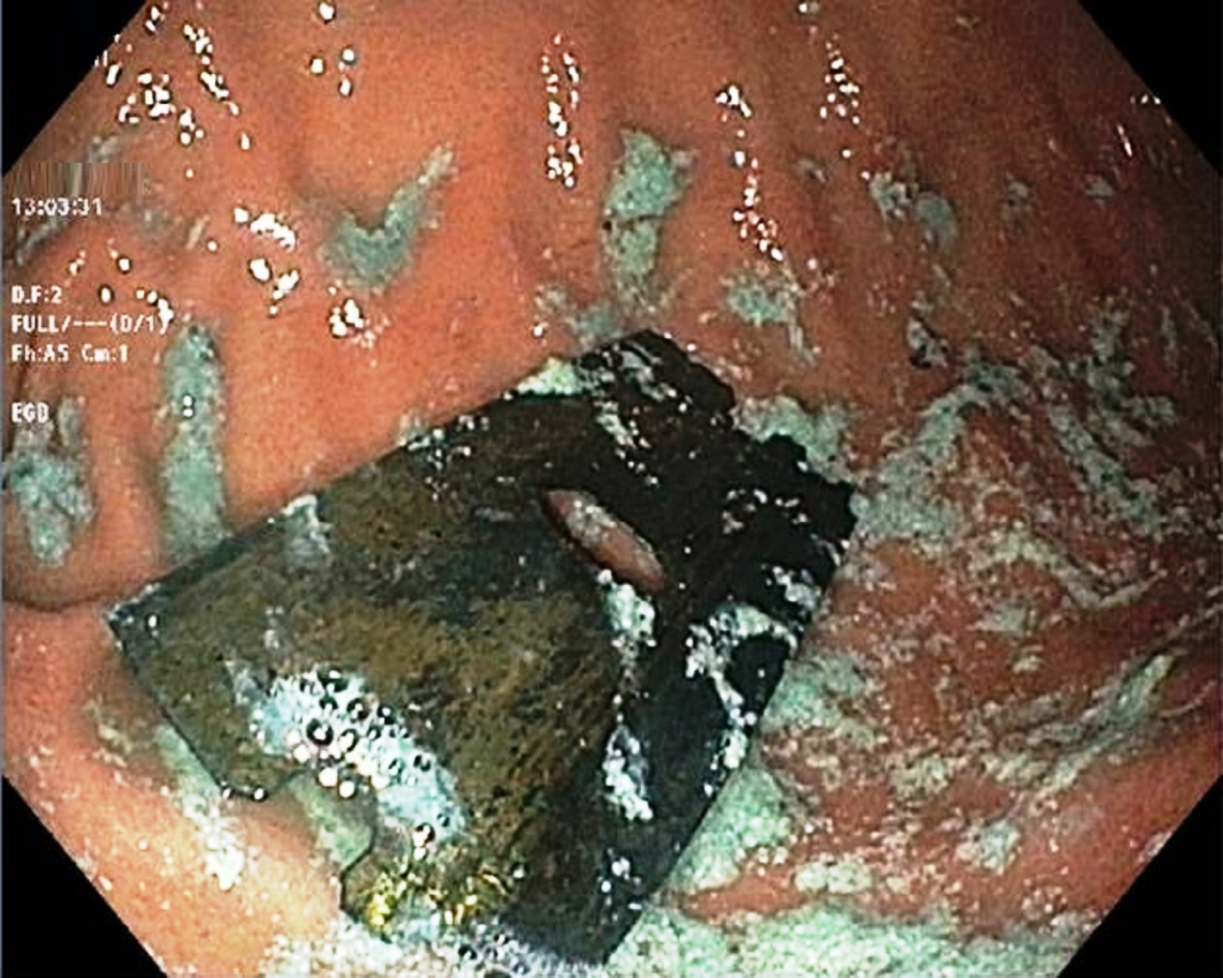
Esophagogastroduodenoscopy showing the razor blade in the body of the stomach with no evidence of mucosal injury or perforation.

Subsequently, urgent esophagogastroduodenoscopy (GIF-H190-2413376; Olympus, Center Valley, PA) was performed, which showed the sharp-edged razor blade in the body of the stomach (Figure 3).

It was easily grasped but was larger (height: 22.0 mm) than the internal diameter (16.7 mm) of the tapered end of the 50-cm long Guardus® overtube (BX00711148; US Endoscopy, Mentor, Ohio). Endoscopy showed minor linear laceration in the cervical esophagus; however, there was no evidence of mucosal injury in the stomach.

**Figure 4:**
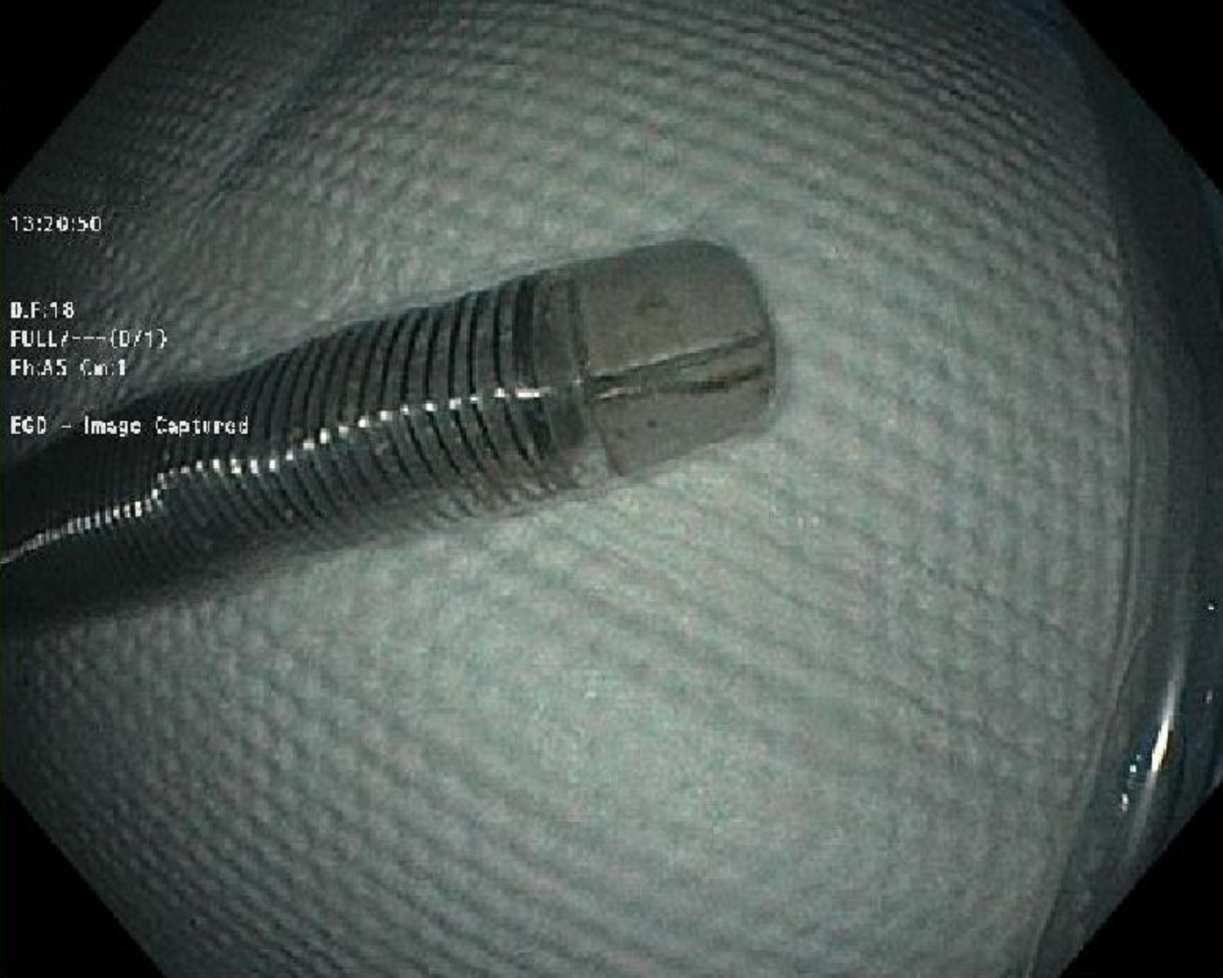
The modification of the overtube provided a flattened distal portion to accommodate the razor blade.

After a consensus of the expert endoscopists, it was decided to modify the overtube. Two small incisions were made at the tapered end followed by flattening the tip of both the outer and inner tubes. This modification resulted in a wider oval shape at the distal end to accommodate the ingested razor blade (Figure 4).

**Figure 5:**
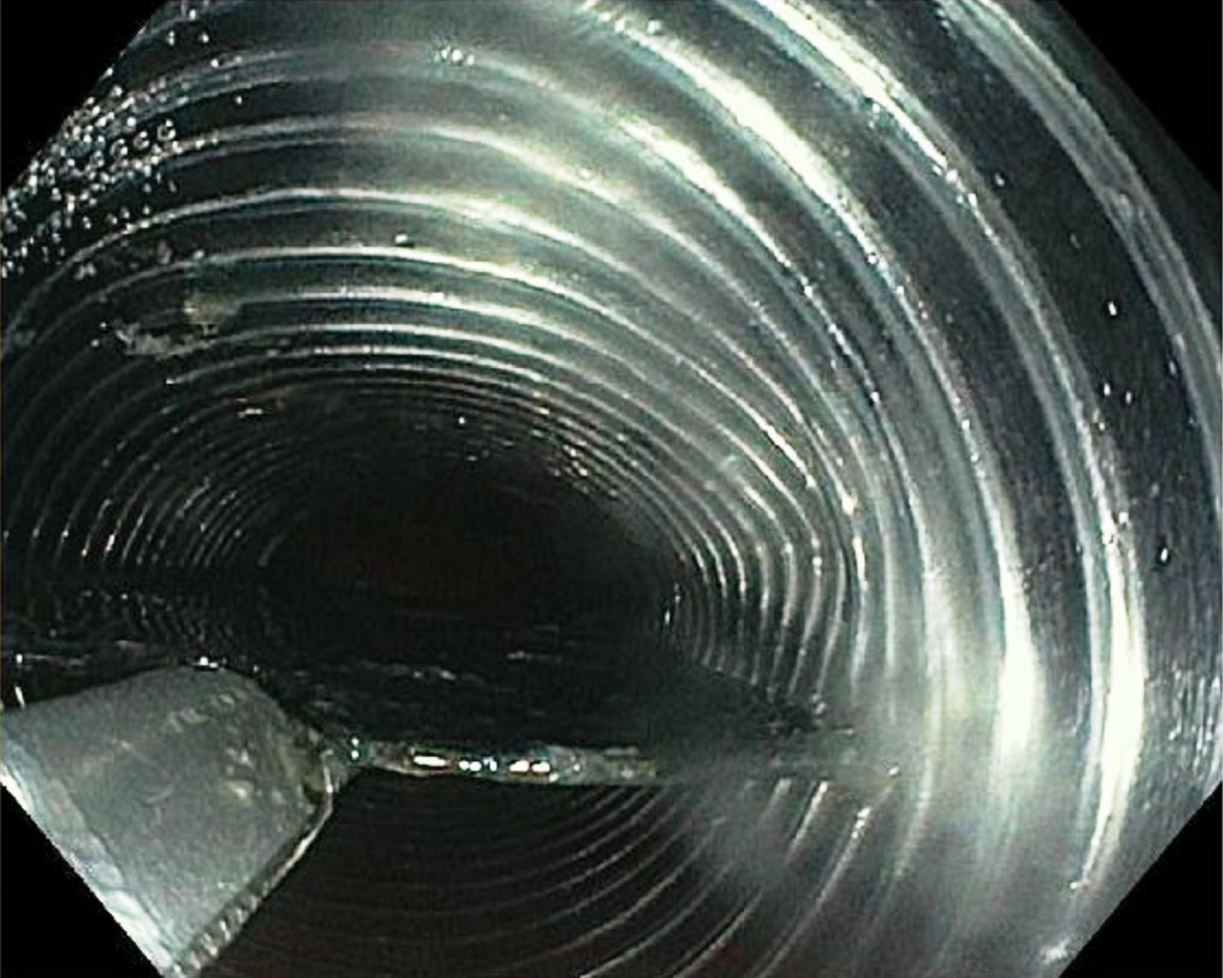
The razor blade was successfully brought into the modified distal flattened portion of the overtube.

The modified overtube system was then backloaded over the endoscope. The razor blade was grasped with a rat-tooth grasper, and it was brought into the distal flattened portion of the overtube (Figure 5).

**Figure 6:**
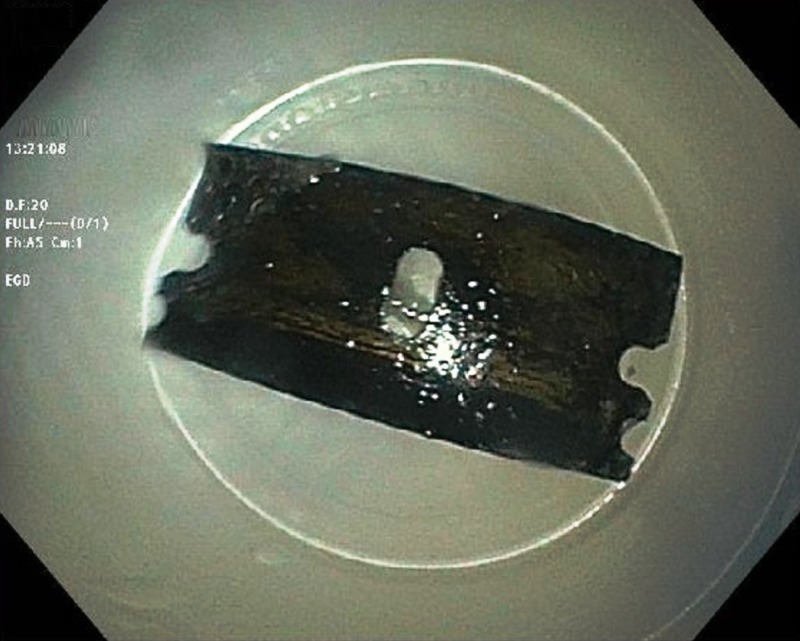
The endoscopic extraction of the razor blade was successful using the modified overtube.

This maneuver resulted in the successful removal of the razor blade (Figure 6).

Re-endoscopy showed no evidence of perforation or injury. Retroflexion was performed in the stomach and the endoscope was withdrawn from the patient. He was transferred back to the surgical intensive care unit. The post-procedure clinical course was uneventful and the patient was transferred to the psychiatric inpatient unit after 24 hours.

The patient showed significant improvement in psychotic symptoms after reinitiation of haloperidol treatment for the schizophrenia relapse. There was no auditory hallucination after treatment and he no longer experienced the urge to ingest objects following commanding auditory hallucination. His speech was coherent and relevant and he was able to hold meaningful conversation. The patient was able to maintain good personal hygiene. He was discharged from the hospital when remission was achieved. In order to avoid potential treatment nonadherence, attempts were made to mobilize family support. Psychoeducation was carried out to help his family understand the need for treatment compliance. At the follow-up psychiatric evaluations, the patient remained in remission for several months now. His level of functioning was also noticeably improved. With improvement of social interactions, social functioning was restored.

## Discussion

Foreign body ingestion is a commonly encountered problem in clinical practice, especially in the pediatric population. True foreign body ingestion in adults is rare and is usually seen in the elderly population, particularly in those with psychiatric disorders, intoxication, developmental delays or in prisoners with secondary gain. About 80–90% of these foreign bodies pass spontaneously while the remaining 10–20% require either endoscopic or surgical removal [1]. The impacted sharp-edged foreign bodies in the gastrointestinal tract are associated with numerous complications such as perforation, mediastinitis, subcutaneous emphysema, aspiration, abscess formation, bleeding, complete bowel obstruction and pressure necrosis resulting in aortic/tracheal fistula formation [2]. Therefore, a prompt diagnosis followed by urgent removal of these objects is of paramount importance.

The diagnostic evaluation includes a detailed history taking that guides about the appropriate imaging modality and indicates the urgency of therapeutic intervention [3]. The best initial screening test is biplane radiography of the affected region. These images provide information about the type, location, size and the number of the foreign bodies. Moreover, they also exclude dangerous complications such as pneumoperitoneum or pneumomediastinum. Plain radiographs identify the majority of objects, except a few radiodense or non-radiodense foreign bodies (e.g., fish or chicken bones, glass, wooden pieces, etc.) where the sensitivity is low (sensitivity, 32%; specificity, 91%) [4]. In these cases, CT scan is preferred (sensitivity, 100%; specificity, 91%). Additionally, CT has a better diagnostic yield if a perforation is suspected. However, inability to detect radiolucent objects is the shortfall of the CT scan, but its sensitivity can be increased by three-dimensional reconstruction [5]. A contrast examination should not be performed as it increases the risk of aspiration and obscures the endoscopic visualization. In this patient, the initial plain abdominal radiograph showed the foreign body likely in the duodenum. However, subsequent CT demonstrated the precise location of the razor blade in the stomach. Although plain radiography is convenient due to its availability and cost-effectiveness, CT scan can be used as an initial diagnostic modality if plain films are expected to be unrevealing [6].

In regards to the management, several retrieval methods have been documented in the literature. The initial management step is to assess the airway, the appropriate timing for intervention and the identification of the suitable retrieval equipment. The usual apparatus comprises of endoscopes, retrieval devices, and overtube. The choice of equipment mostly depends on the location of the foreign body. If it is located at or above the cricopharyngeus, the direct laryngoscope is used. If retrieval fails or the foreign body is positioned below the cricopharyngeus, flexible or rigid endoscopes can be used [7]. Flexible endoscopes are preferred over rigid ones due to the higher success rate and lower perforation risk [8]. For proximal foreign bodies, rigid endoscopic removal can be undertaken without the use of overtube. Among the retrieval devices, rat-tooth forceps, alligator forceps, polypectomy snares, polyp graspers, Dormier baskets, retrieval nets, magnetic probes, friction-fit adaptors and/or banding caps have frequently been employed. Polypectomy snares are the most useful for sharp objects [9]. Few unique extraction methods have also been documented in the published literature. Hiller and Hagberg described a case where the sharp object was long and did not fit into the overtube. The use of video laryngoscope adjunct to the flexible endoscopes was helpful as it reduced the risk of perforation and extraction failure [10]. Similarly, Kim et al. described a case where double-balloon enteroscopy was used for retrieval of a screw nail in the jejunum [11]. In most cases with sharp foreign bodies, an overtube has been commonly used in order to reduce the risk of procedure-related complications [12].

An overtube is a semi-rigid plastic sleeve-like device with its inner diameter similar to that of an endoscope. Although overtubes vary in length (23–135 cm) and diameter (14–21 mm), a minimum length of 50 cm is required to ensure the protection of both the esophagus and the stomach [13]. The inner diameter of a standard Guardus overtube is 16.7 mm, and the outer diameter is 19.5 mm. The minimum esophageal length of a standard overtube is 25 cm. An overtube protects the esophageal mucosa and provides a passage for quickly repeated intubations [14]. The insufflation cap helps maintain insufflation and guards against the leakage of body fluids. While grasping a sharp object with a standard overtube, expert endoscopists keep the sharp end of the object trailing distally to avoid any mucosal injury. Furthermore, the long objects such as razor blades should be held from the end instead of the center. Holding the object at the center turns it lengthwise across the lumen and prevents it from being pulled across the sphincters and the esophagus. Although most cases can be salvaged using the standard endoscopic equipment, it may become difficult to remove sharp-edged foreign bodies in few instances, especially when the size of the object is considerably large.

According to our literature review, this report represents the second case describing the removal of the sharp-edged razor blade with the utilization of this novel retrieval process. This case is remarkable for a standard overtube's ability to accommodate a sharp-edged foreign body larger than its diameter after a unique modification. The height of the blade was larger (22 mm) than the internal diameter of the Guardus overtube (16.7 mm). However, with the modification of the tip of the overtube after giving two small incisions and making the distal end flat, the razor blade was removed successfully. Alhaji et al.reported a similar case where the patient had bipolar disorder and ingested a razor blade [15]. Esophagogastroduodenoscopy revealed the foreign body in the esophagus. It was easily grasped but did not fit into the internal diameter of the overtube (16.7 mm). The patient was taken to the operating room where plain radiograph showed the migration of the blade to the stomach. The distal 2 mm of the overtube was incised and an oval flap was created to retrieve the object successfully. Hence, it is a safe and useful method to retrieve the sharp-edged objects, particularly if their dimensions exceed the internal diameter of the overtube.

## Conclusions

Impacted sharp-edged objects in the gastrointestinal tract can be challenging to manage, with different methods described in the literature for their safe removal. This report illustrates an unusual modification of a commercial overtube to safely remove a sharp foreign body that would not otherwise have been possible with the standard equipment available.

## References

[1] Birk M, Bauerfeind P, Deprez PH (2016). Removal of foreign bodies in the upper gastrointestinal tract in adults: ESGE Clinical Guideline. Endoscopy.

[2] ASGE Standards of Practice Committee, Ikenberry SO, Jue TL (2011). Management of ingested foreign bodies and food impactions. Gastrointest Endosc.

[3] Zhang X, Jiang Y, Fu T, Zhang X, Li N, Tu C (2017). Esophageal foreign bodies in adults with different durations of time from ingestion to effective treatment. J Int Med Res.

[4] Lee JH, Kim HC, Yang DM, Kim SW, Jin W, Park SJ, Kim HJ (2012). What is the role of plain radiography in patients with foreign bodies in the gastrointestinal tract?. Clin Imaging.

[5] Takada M, Kashiwagi R, Sakane M, Tabata F, Kuroda Y (2000). 3D-CT diagnosis for ingested foreign bodies. Am J Emerg Med.

[6] Marco De Lucas E, Sadaba P, Lastra Garcia-Baron P, Ruiz-Delgado ML, Gonzalez Sanchez F, Ortiz A, Pagola MA (2004). Value of helical computed tomography in the management of upper esophageal foreign bodies. Acta Radiol.

[7] Geraci G, Sciume' C, Di Carlo G, Picciurro A, Modica G (2016). Retrospective analysis of management of ingested foreign bodies and food impactions in emergency endoscopic setting in adults. BMC Emerg Med.

[8] Gmeiner D, von Rahden BH, Meco C, Hutter J, Oberascher G, Stein HJ (2007). Flexible versus rigid endoscopy for treatment of foreign body impaction in the esophagus. Surg Endosc.

[9] Tang SJ (2013). Endoscopic management of foreign bodies in the gastrointestinal tract. Video J Encycl GI Endosc.

[10] Hiller KN, Hagberg CA (2016). Use of a video laryngoscope to facilitate removal of a long, sharp-pointed blade from the esophagus. J Clin Anesth.

[11] Kim DJ, Sim MK, Lee SW, Lee TH (2015). Successful removal of a screw nail in the jejunum using double-balloon enteroscopy. Clin Endosc.

[12] Smith MT, Wong RK (2007). Foreign bodies. Gastrointest Endosc Clin N Am.

[13] Wells CD, Fleischer DE (2008). Overtubes in gastrointestinal endoscopy. Am J Gastroenterol.

[14] Tierney WM, Adler DG, Conway JD (2009). Overtube use in gastrointestinal endoscopy. Gastrointest Endosc.

[15] Alhaji M, Atreja A, Upchurch BR (2009). Razor blade removal from the stomach utilizing a novel modification of the overtube. Endoscopy.

